# The weight of nations: an estimation of adult human biomass

**DOI:** 10.1186/1471-2458-12-439

**Published:** 2012-06-18

**Authors:** Sarah Catherine Walpole, David Prieto-Merino, Phil Edwards, John Cleland, Gretchen Stevens, Ian Roberts

**Affiliations:** 1 Foundation Year 2 doctor, North Yorkshire and East Coast deanery, 4 Hilton Place, Leeds, LS8 4HE, UK; 2 Faculty of Epidemiology and Population Health, London School of Hygiene & Tropical Medicine, Keppel Street, London, WC1E 7HT, UK; 3 Department of Health Statistics and Informatics, World Health Organization, 20 Avenue Appia, Geneva 27, 1211, Switzerland

## Abstract

**Background:**

The energy requirement of species at each trophic level in an ecological pyramid is a function of the number of organisms and their average mass. Regarding human populations, although considerable attention is given to estimating the number of people, much less is given to estimating average mass, despite evidence that average body mass is increasing. We estimate global human biomass, its distribution by region and the proportion of biomass due to overweight and obesity.

**Methods:**

For each country we used data on body mass index (BMI) and height distribution to estimate average adult body mass. We calculated total biomass as the product of population size and average body mass. We estimated the percentage of the population that is overweight (BMI > 25) and obese (BMI > 30) and the biomass due to overweight and obesity.

**Results:**

In 2005, global adult human biomass was approximately 287 million tonnes, of which 15 million tonnes were due to overweight (BMI > 25), a mass equivalent to that of 242 million people of average body mass (5% of global human biomass). Biomass due to obesity was 3.5 million tonnes, the mass equivalent of 56 million people of average body mass (1.2% of human biomass). North America has 6% of the world population but 34% of biomass due to obesity. Asia has 61% of the world population but 13% of biomass due to obesity. One tonne of human biomass corresponds to approximately 12 adults in North America and 17 adults in Asia. If all countries had the BMI distribution of the USA, the increase in human biomass of 58 million tonnes would be equivalent in mass to an extra 935 million people of average body mass, and have energy requirements equivalent to that of 473 million adults.

**Conclusions:**

Increasing population fatness could have the same implications for world food energy demands as an extra half a billion people living on the earth.

## Background

Thomas Malthus’ Essay on the Principle of Population warned that population increase would eventually outstrip food supply, resulting in famine [1]. Malthus expressed his concern at a time when the amount of food energy that could be harvested from a given amount of land was constrained by the available agricultural technologies. The Green Revolution of the twentieth century challenged Malthus’ grim predictions, as fossil fuel-based fertilizers, pesticides, irrigation and mechanization greatly increased food yields [2]. In the twenty first century, the link between population and ecological sustainability is again coming to the fore, as global food yields are threatened by ecological destruction (including climate change) and as world population grows [2].

The energy requirement of species at each trophic level in an ecological pyramid is a function of the number of organisms and their average mass. In ecology, these factors are often considered together by estimating species biomass, the total mass of living organisms in an ecosystem. In relation to human populations, although much attention is given to the effect of population growth on food energy requirements, much less attention is given to the impact of increasing body mass.

Physical activity accounts for 25-50% of human energy expenditure. Due to the greater energy cost of moving a heavier body, energy use increases with body mass [3]. Resting energy expenditure also increases with body mass, due to the increase in metabolically active lean tissue that accompanies increases in body fat [4]. As for other organisms, the energy requirements of human populations depend on species biomass. Currently, more than a billion adults are overweight and in all regions of the world,, the entire population distribution of body mass is moving upwards [5].

The increased global demand for food arising from the increase in body mass is likely to contribute to higher food prices. Because of the greater purchasing power of more affluent nations (who also have higher average body mass), the worst effects of increasing food prices will be experienced by the world’s poor. In this article, we estimate total human biomass, its distribution by world region and the proportion of human biomass attributable to overweight and obesity.

## Methods

### Data sources

For each country, we obtained estimates of the population in 2005 by age and sex from the United Nations population database [6]. We obtained estimates of mean (and SD) body mass index (BMI) from the *WHO SURF2* report [7] and estimates of mean height (and SD) for 190 countries from national health examination surveys, primarily the *Demographic and Health Surveys*[5]. Because surveys were not conducted in every country, height data were not available by age and sex in some countries. To estimate mean height (and SD) by age and sex in every country using the available data, we built a linear regression model (of age-sex group, average height, WHO region and sub-region) using *R* open access statistical software. Some countries and territories were excluded from the analysis due to insufficient data on BMI (see Table 1 for a list of these).

**Table 1:** List of excluded countries due to insufficient data on BMI

**Country / Territory**	**UN code**	**Adult pop. (2005)**
Other non-specified areas (Taiwan)	158	18,405,317
Serbia	688	8,037,649
China, Hong Kong SAR	344	5,840,953
Puerto Rico	630	2,936,606
Occupied Palestinian Territory	275	1,928,679
Réunion	638	582,423
Montenegro	499	502,268
China, Macao SAR	446	401,495
Guadeloupe	312	338,621
Martinique	474	313,280
Western Sahara	732	301,959
French Polynesia	258	185,626
New Caledonia	540	168,610
Netherlands Antilles	530	143,172
French Guiana	254	130,255
Channel Islands	830	124,942
Guam	316	119,046
Mayotte	175	101,272
United States Virgin Islands	850	84,706
Aruba	533	79,238
TOTAL:		40,726,117

Formula for estimating the expected (average) weight (W) in a specific age-sex group where mean and variance of BMI and of height.

Using the following notation for each individual values of BMI and W: $b=\left(BMI-\bar{BMI}\right)\;\text{and}\;h=\left(H-\bar{H}\right)$

The expected weight in a group of individuals would be: $\begin{array}{l} E\left(W\right)=E\left(BMI\times H^{2}\right)=E\left(\left(\bar{BMI}+b\right)\times {\left(\bar{H}+h\right)}^{2}\right)=E\left(\left(\bar{BMI}+b\right)\times \left({\bar{H}}^{2}+h^{2}+2\bar{H}h\right)\right)\\ =E\left({\bar{H}}^{2}\bar{BMI}+h^{2}\bar{BMI}+2\bar{H}h\bar{BMI}+{\bar{H}}^{2}b+h^{2}b+2\bar{H}hb\right)\\ ={\bar{H}}^{2}\bar{BMI}+E\left(h^{2}\right)\bar{BMI}+E\left(h\right)2\bar{H}\bar{BMI}+E\left(b\right){\bar{H}}^{2}+E\left(bh^{2}\right)+E\left(hb\right)2\bar{H}\\ ={\bar{H}}^{2}\bar{BMI}+E\left(h^{2}\right)\bar{BMI}+E\left(bh^{2}\right)+E\left(hb\right)2\bar{H} \end{array}$

Assuming that Height and BMI are independent: $COV(H,BM)=0\Rightarrow E\left(hb\right)=0$

Assuming that the variance of Height is constant in all values of BMI: $E\left(bh^{2}\right)=0$

Therefore the above equation simplifies to: $\;E\left(W\right)=\bar{BMI}\times \left({\bar{H}}^{2}+V(H)\right)$

### Biomass estimation

Total biomass by age-sex group was estimated as the product of the number of people in the group and their average body mass. The formulae for the estimation of body mass are given in the appendix. We also estimated total biomass due to overweight in each age-sex group. We assumed that BMI is normally distributed in the group and estimated the number of people overweight (using prevalence of BMI > 25) and their average BMI. Using their average BMI, we then estimated their average body mass. The biomass of overweight people was calculated as the product of the number of overweight people and their average body mass. Biomass due to overweight was calculated by estimating the biomass of overweight people assuming they had BMI of 25 and subtracting this from their actual biomass. Using a similar method we estimated the biomass due to obesity. We calculated the total biomass of obese people in each age-sex group and subtracted their estimated biomass assuming that they all had a BMI of 30. For each country, we calculated total human biomass, biomass due to overweight and biomass due to obesity by adding the estimates for each age-sex group. Global totals were calculated by summating across countries.

### Extreme case scenarios

We estimated global biomass under two hypothetical scenarios. Specifically, we assumed that each country had the same BMI distributions as that of [1] Japan and [2] USA. We used the method outlined above but applied the BMI of the relevant age-sex group from Japan or USA instead of the actual BMI for that age-sex group. These countries were chosen because despite being high income countries with adequate nutrition, they have average BMI values close to global extremes. For each scenario, we calculated the global biomass and biomass due to overweight and obesity.

### Population and energy equivalents

We calculated the food energy required to sustain human biomass using formulae and values from the FAO [8]. Physical Activity Level (PAL) values for each age-sex group are based on non-overweight adults in the USA. Total Energy Expenditure (TEE) is estimated as the product of Basal Metabolic Rate (BMR) and PAL (see Table 2). The energy required to sustain the biomass due to overweight, obesity or the change in biomass that would be seen under hypothetical scenarios, was estimated by multiplying the number of kg by weight dependent component of BMR and by the PAL. We did all calculations by country and age-sex group applying the corresponding coefficients. Then we summed across age-sex groups to obtain total energy requirements for each country and for the world. To calculate the number of average adults that could be sustained with a given quantity of biomass we divided the amount of energy required to sustain that biomass by the average food energy requirement of one human.

**Table 2:** Estimation of Basal Metabolic Rate (BMR) and Total Energy Expenditure (TEE) using FAO tables

		**Men**			**Women**	
	BMRc	BMRs	PAL(*)	BMRc	BMRs	PAL(*)
age	Kcal	Kcal/kg		Kcal	Kcal/kg	
15-29	692.2	15.057	1.75	486.6	14.818	1.79
30-44	873.1	11.472	1.82	845.6	8.126	1.87
45-59	873.1	11.472	1.64	845.6	8.126	1.8
60-69	587.7	11.171	1.61	658.5	9.082	1.69
70-79	587.7	11.171	1.62	658.5	9.082	1.55
80+	587.7	11.171	1.3	658.5	9.082	1.19

We extracted the following coefficients for our age-sex groups.

(*) For non overweight adults in USA.

The estimation of energy requirements of an individual is: $\text{BMR }=\text{ BMRc }+\text{ BMRs x Weight}\left(\text{kg}\right)\to \text{TEE }=\text{ BMR x PAL}$

For a group of N individuals of the same age-sex group with a total biomass “B”, $\text{BMR }=\text{ N x BMRc }+\text{ BMRs x BM}\left(\text{kg}\right)\to \text{TEE }=\text{ BMR x PAL}$

If that same group had a biomass due to overweight (BMI > 25) of “B25”, the energy required to feed that biomass would be: $\text{BMR25 }=\text{ BMRs x B}25\left(\text{kg}\right)\to \text{TEE25 }=\text{ BMR25 x PAL}$

## Results

In 2005, total adult human biomass was approximately 287 million tonnes (Table 3). Biomass due to overweight was 15 million tonnes, the mass equivalent of 242 million people of average body mass (approximately 5% of the world’s population in 2005). Biomass due to obesity was 3.5 million tonnes, the mass equivalent of 56 million people of average body mass (1.2% of the world’s population). Average body mass globally was 62 kg.

**Table 3:** Population, body mass and biomass by world region in 2005 and in hypothetical scenarios

**WHO region**	**Adult population (millions)**	**Average body mass (kg)**	**Biomass (million kg)**	**No of people overweight / total population**	**Biomass due to BMI > 25 (million kg)**	**Biomass due to BMI > 30 (million kg)**
Asia	2815	57.7	162408	24.2%	4265	449
Europe	606	70.8	42895	55.6%	3836	910
Africa	535	60.7	32484	28.9%	1464	340
Latin Am. Caribbean	386	67.9	26231	57.9%	2431	585
Northern Am.	263	80.7	21185	73.9%	3297	1187
Oceania	24	74.1	1815	63.3%	191	46
World	4630	62.0	287017	34.7%	15484	3518
Scenario (1): all countries have BMI distribution of Japan	4630	58.8	272408 (−5%)	22.3%	5630 (−64%)	253 (−93%)
Scenario (2): all countries have BMI distribution of USA	4630	74.6	345426 (+20%)	74.0%	53090 (+243%)	18789 (+434%)

North America has the highest average body mass of any continent (80.7 kg). In North America one tonne of human biomass corresponds to 12 adults. More than 70% of the North American population is overweight and biomass due to obesity is 1.2 million tonnes. North America has 6% of the world’s population but 34% of world biomass due to obesity. Asia has the lowest average body mass of any continent (57.7 kg). In Asia, one tonne of human biomass corresponds to 17 adults. Asia has 61% of the world’s population but 13% of world biomass due to obesity (449 thousand tonnes).

The average BMI in Japan in 2005 was 22.9. If all countries had the same age-sex BMI distribution as Japan, total biomass would fall by 14.6 million tonnes, a 5% reduction in global biomass or the mass equivalent of 235 million people of world average body mass in 2005. This reduction in biomass would decrease energy requirements by an average of 59 kcal/day per adult living on the planet, which is equivalent to the energy requirement of 107 million adults. Biomass due to obesity would be reduced by 93%.

The average BMI in USA in 2005 was 28.7. If all countries had the same age-sex BMI distribution as the USA, total human biomass would increase by 58 million tonnes, a 20% increase in global biomass and the equivalent of 935 million people of world average body mass in 2005. This increase in biomass would increase energy requirements by 261 kcal/day/adult, which is equivalent to the energy requirement of 473 million adults. Biomass due to obesity would increase by 434%.

**Figure 1:**
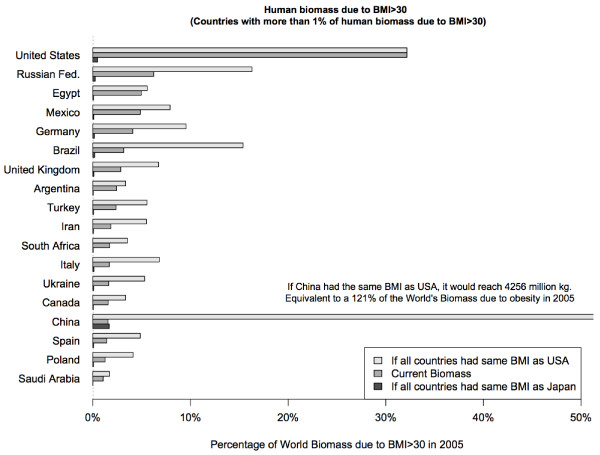
Human biomass due to BMI > 30 (Countries with more than 1% of human biomass due to BMI > 30).

Figure 1 shows the distribution of biomass due to obesity for countries with more than 1% of total human biomass. The two scenarios are also reflected. If China had the same BMI distribution as the USA its biomass due only to obesity would be equivalent to 121% of the world total of biomass due to obesity in 2005.

The energy required to maintain obese biomass corresponds to the energy requirements of 24 million adults of world average body mass (Table 4). The energy required to maintain overweight biomass corresponds to the energy requirements of 111 million average adults. In the United States alone, the energy required to maintain overweight biomass corresponds to the energy requirements of 23 million adults of world average body mass (Table 4). If all countries had the same BMI distribution as USA, the energy required to maintain obese biomass would increase by 481%, corresponding to the energy requirements of 137 million adults. Under this scenario, the energy required to maintain overweight biomass corresponds to the energy requirements of 406 million adults.

**Table 4:** Adults per tonne biomass and energy used to maintain overweight and obesity

**Country with more than 100,000 population**	**Adults per tonne**	**Average tTEE**[**1**]**Kcal/day/ adult**	**Average energy used to maintain biomass due to BMI > 25 in kcal/day/adult**	**Thousands of adults of average mass that could be maintained by the energy required to maintain BMI >25 BMI >30**	
*Heaviest 10*					
United States	12.2	2874	243	22,509.2	7,886.6
Kuwait	12.9	2982	233	156.6	53.9
Croatia	13.1	2741	205	300.3	96.0
Qatar	13.0	3007	204	51.6	14.5
Egypt	13.5	2826	192	3,733.5	1,184.2
United Arab Emirates	13.2	3017	188	241.2	62.8
Trinidad and Tobago	13.8	2778	177	71.3	21.7
Argentina	13.8	2718	176	1,967.9	575.7
Greece	13.3	2707	169	636.0	159.3
Bahrain	13.6	2889	168	34.8	9.7
*Lightest 10*					
North Korea	19.0	2348	8	57.5	1.5
Cambodia	17.9	2472	7	23.9	0.2
Burundi	18.5	2421	7	11.4	0.4
Nepal	19.8	2354	7	42.4	0.6
Democ. Rep. of the Congo	18.7	2410	6	71.2	2.2
Bangladesh	20.2	2342	5	178.2	2.7
Sri Lanka	19.8	2318	5	27.5	0.3
Ethiopia	18.9	2408	3	52.9	0.5
Viet Nam	19.7	2341	3	73.7	1.1
Eritrea	19.2	2393	2	2.0	0.0
WORLD 2005	16.1	2549	61	111,346	23,533
Scenario (1) if BMI as Japan in all countries	17.0	2490 (−2.4%)	22	40,519 (−64%)	1,726 (−93%)
Scenario (2) if BMI as USA in all countries	13.4	2810 (+10.2%)	224	406,255 (+265%)	136,721 (+481%)

(1) tTEE = theoretical Total Energy Expenditure calculated from FAO tables for adults, assuming that Physical Activity Levels (PAL) for each age-sex group in all countries were the same as those reported for USA in the same document. (2) To calculate these two columns we use the average theoretical tTEE of the world in 2005 (2549 kcal/day).

## Discussion

We estimated global human biomass, its regional distribution and biomass attributable to overweight and obesity. Our results underscore the need to take body mass into account when considering the ecological implications of population growth. UN world population projections suggest that by 2050 there could be an additional 2.3 billion people. [6] The ecological implications of rising population numbers will be exacerbated by increases in average body mass.

Although the largest increase in population numbers is expected in Asia and sub-Saharan Africa, our results suggest that population increases in the USA will carry more weight than would be implied by numbers alone. It is predicted that the US population will increase from 310 million in 2010 to 403 million by 2050 [5]. Most of the increase will be due to migration and to the extent that migrants adopt the diet and lifestyles of the host population, we can reasonably expect that the body mass of migrants will rise. Our results show that this could have important implications for world energy requirements.

In Africa and Asia urban populations are increasing more rapidly than rural populations [9]. This will also have implications for average body mass [10]. Given the current trend of rising BMI, our scenario where all countries have a similar BMI distribution to the USA provides an insight into possible future challenges. If global biomass were to increase to a level where all countries had the age-sex BMI distributions of the USA, the biomass increase would be equivalent to an extra billion people of average body mass. Although, this is not the same as an extra billion people in terms of energy requirements, the increase corresponds to the energy requirements of about 473 million adults of current world average body mass.

Our findings should be viewed in the light of the following limitations. Firstly, in countries where data on average BMI, height and its standard deviation were unavailable, we used a regression model to estimate the missing parameters. Due to limited data availability, we assumed that height and BMI are independent variables, and that the mean and standard deviation of height are the same across the distribution of BMI. Furthermore, because of the lack of data describing the distribution of BMI in relation to high, we assumed zero covariance between BMI and height squared. Secondly, we assumed symmetrical (normal) distributions of BMI in each population, when in reality many population distributions will be skewed, with a tail to the right of the distribution comprising a relatively small proportion of people with very high body mass. We may therefore have underestimated total biomass. Finally, we did not estimate biomass in children who comprise a significant proportion of the population in many countries, nor in countries with population less than 100,000. Future work in this area should account for population age structure, as well as education levels and urbanisation.

There are also limitations in our estimates of energy requirements. We have used FAO data to estimate the BMR but the extent to which they can be applied to all populations is open to question. The assumption of similar physical activity levels in all countries is clearly unrealistic with higher physical activity levels in low income countries. As a result, we will have underestimated energy requirements in some countries. However, this approach is appropriate for comparing different scenarios of BMI distribution and its implications on relative changes in energy requirements.

## Conclusions

Increasing biomass will have important implications for global resource requirements, including food demand, and the overall ecological footprint of our species. Future work will investigate the extent to which food demand and carbon emissions are likely to increase with increasing biomass.

Although the concept of biomass is rarely applied to the human species, the ecological implications of increasing body mass are significant and ought to be taken into account when evaluating future trends and planning for future resource challenges. Our scenarios suggest that global trends of increasing body mass will have important resource implications and that unchecked, increasing BMI could have the same implications for world energy requirements as an extra 473 million people. Tackling population fatness may be critical to world food security and ecological sustainability.

## Competing interests

The authors declare that they have no competing interests.

## Authors’ contributions

GS is a staff member of WHO. The author alone is responsible for the views expressed in this publication and they do not necessarily represent the decisions, policy, or views of WHO. IR devised the study; SW, DP and PE conducted the analyses with input from GS; and all authors contributed to writing and revising the manuscript. All authors read and approved the final manuscript.

## Pre-publication history

The pre-publication history for this paper can be accessed here:

http://www.biomedcentral.com/1471-2458/12/439/prepub
